# Effects of endogenous and exogenous micronutrients in rapeseed oils on the antioxidant status and lipid profile in high-fat fed rats

**DOI:** 10.1186/1476-511X-13-198

**Published:** 2014-12-19

**Authors:** Qianchun Deng, Xiao Yu, Jiqu Xu, Lan Wang, Fenghong Huang, Qingde Huang, Changsheng Liu, Fangli Ma

**Affiliations:** Oil Crops Research Institute, Chinese Academy of Agricultural Sciences, 2 Xudong Second Road, Wuhan, 430062 China; Hubei Key Laboratory of Lipid Chemistry and Nutrition, Wuhan, 430062 China; Institute for Farm Products Processing and Nuclear-Agricultural Technology, Hubei Academy of Agricultural Science, Wuhan, 430064 China; Functional Oil Laboratory Associated by Oil Crops Research Institute, Chinese Academy of Agricultural Sciences and Infinite (China) Co., LTD, Guangzhou, 51000 China

**Keywords:** Rapeseed oil, Cold-pressed, Tocopherols, Phytosterols, Antioxidant status

## Abstract

**Background:**

Micronutrients in oil reduce one or more risk factors of cardiovascular diseases, while the contents of micronutrients in oil are relatively poor, which is insufficient to reverse the metabolic disorders at different stages of progress. The aim of this study was to investigate the effects of endogenous micronutrients in optimized cold-pressed rapeseed oil and restoratively added or fortified micronutrients in traditional refined rapeseed oil (restoring micronutrients to be nearly equal to or significantly higher than levels in crude rapeseed oil) on the antioxidant status and lipid profile in high-fat fed rats.

**Methods:**

Male Wistar rats were fed high-fat diets containing different rapeseed oils for 4 weeks, including the standard refined rapeseed oil(SRO), optimized cold-pressed rapeseed oil(CRO) and the traditional refined rapeseed oil with restorative addition or fortification of micronutrients (LF, HF-SRO).

**Results:**

CRO exhibited significant increases in contents of tocopherols (+13%), phytosterols (+34%), polyphenols (+92%) and phospholipids (+725%) compared with SRO, as well as the total antioxidant capacities (+82-125%) (p < 0.05). While the HF-SRO revealed improved antioxidant properties *in vitro* than the CRO, which was comparable to LF-SRO. Significant improved plasma antioxidant capacities and lipid peroxidation evaluated by T-AOC, GSH, tocopherols and MDA were found in rats fed HF-SRO when compared with CRO and LF-SRO (p < 0.05). Furthermore, HF-SRO also decreased the plasma and hepatic TC levels compared to CRO and LF-SRO, accompanying higher fecal cholesterol excretion (p < 0.05).

**Conclusion:**

The standard refined rapeseed oil with fortification, not restorative addition of micronutrients was comparable to the optimized cold-pressed rapeseed oil in improving the antioxidant status and lipid profile of high-fat fed rats.

## Background

Rapeseed (*Brassica napus L.*) is the second most important oilseed crop in the world after soybean, and the consumption of rapeseed and its products have been increasing in human diets throughout the world in part due to the recognized importance of high levels of polyunsaturated fatty acids (PUFAs) and favorable ratio of n-6 to n-3 fatty acids. Rapeseed oil also contains different levels of tocopherols (8–115.9 mg/100 g), phytosterols (459–5000 mg/100 g), polyphenols (0–148.7 mg/100 g) and other micronutrients for variety, maturity and processing technology specificities [1]. And more recent studies in animals and humans have demonstrated the bioactivity of micronutrients (phytosterols, tocopherols, phospholipids, phenols and CoQ_9_+ CoQ_10_) from rapeseed oil in reducing the risk factors of cardiovascular diseases by significantly improving the triglyceride and cholesterol profiles, reducing LDL oxidation and optimizing the endogenous antioxidant status [2–4]. Whereas the classic oil processing and refining technologies in order to remove primary and secondary oxidation products and other harmful components in crude oil have been shown to remove 26–55% and 99% of phenolic and carotenoid compounds followed by 80% loss of antioxidant capacity in palm oil, and 34% of tocopherols, 26% of free phytosterols and almost complete removal of polyphenols in rapeseed oil [5, 6]. Therefore, the traditional technologies for oil processing should be optimized for better recovery of micronutrients. Currently, some optimized processing technologies that favorably preserve higher contents of bioactive compounds have been studied, including cooking and pressing after dehulling or flaking, hexane extraction after cold-pressed procedure, and ethanol extraction after twin-screw extrusion [2–4, 7].

However, more work is still needed for these optimized technological procedures to be successfully applied to the practical production, such as the low yield in cold-pressed oil and solvent residues in solvent selectively extracted oil as well as the strict requirements for the raw material quality due to no further refining process. In addition, the contents of micronutrients in oil are relatively poor (*ppm* levels), which may be insufficient to ameliorate the metabolic disorders at different stages of progress. Considering from the cutting edge between dietary and pharmacological dose of these minor compounds, artificially adding micronutrients to the traditional refined oils may be another expedient method. Thus, it is necessary to investigate whether the restoratively added or fortified micronutrient (restoring micronutrients to be nearly equal to or significantly higher than levels in crude rapeseed oil) could exert similar bioactivity in preventing several metabolic pathologies as the micronutrients naturally present in oil.

The present study compared the effects of optimized cold-pressed rapeseed oil with endogenous bioactive components and traditional refined rapeseed oil with restoratively added or fortified exogenous tocopherols and phytosterols (the main antioxidant compounds in rapeseed oil) on the antioxidant status and lipid profile in high-fat fed rats, which could provided a theoretical basis for obtaining the rapeseed oil, to some extent remedied the adverse effect of traditional processing technology.

## Results

### Physicochemical and antioxidant characteristics *in vitro*of different rapeseed oils

As reported in Table 1, there were no significant differences for the major fatty acid composition among the different rapeseed oils. However, the *trans* fatty acid concentration was nearly 4-fold increases in SRO compared to CRO, especially the levels of *trans*-linolenic acid (C18:3 *trans*) (p < 0.05). CRO retained the free fatty acid and moisture from raw material and therefore exhibited higher acid value and moisture contents than that in SRO for no further traditional refining process (1.14 mg KOH/g vs.0.15 mg KOH/g; 0.087% vs.0.057%) (p < 0.05). A great effect of traditional processing technology on the micronutrient contents in rapeseed oil was revealed in this study. The tocopherols, phytosterols, polyphenols and phospholipids contents in CRO increased by 13%, 34%, 92% and 725%, respectively, when compared with SRO (p < 0.05). The quality parameters of LF- and HF-SRO were similar to that of SRO except for the exogenous phytosterols and tocopherols contents. The oil stability index (OSI) was performed to predict and compare the expected oil stability by treating the oil in increased oxidation conditions. SRO was more stable upon the OSI treatment compared to CRO (p < 0.05). While LF- and HF-SRO exhibited higher values of OSI than SRO accompanied by the exogenous addition of phytosterols and tocopherols, and there were also significant differences between the HF-SRO and CRO (p < 0.05).

**Table 1:** **Quality characteristics and total antioxidant activity of different rapeseed oils**

FA (%)	SRO	CRO	LF-SRO	HF-SRO
C16:0	3.78	3.70	3.78	3.78
C16:1 *trans*	0.04	0.03	0.04	0.04
C16:1 *cis*	0.20	0.19	0.20	0.20
C18:0	2.43	2.00	2.43	2.43
C18:1 *trans*	0.09	0.11	0.09	0.09
C18:1 *cis*	62.78	66.43	62.78	62.78
C18:2 *trans*	0.19	ND	0.19	0.19
C18:2 *cis*	17.36	17.02	17.36	17.36
C18:3 *trans*	0.83	ND	0.83	0.83
C18:3 *cis*	6.93	7.73	6.93	6.93
∑SFA	6.21	5.70	6.21	6.21
∑MUFA	63.11	66.76	63.11	63.11
∑PUFA	25.31	24.75	25.31	25.31
Total trans fatty acids	1.15 ± 0.02	0.33 ± 0.01*	1.15 ± 0.02^#^	1.15 ± 0.02^#^
Acid Value (mgKOH/g)	0.15	1.14	0.15	0.15
Peroxide value (meq/kg)	1.19	1.38	1.19	1.19
Water and volatile matter (%)	0.057 ± 0.002	0.087 ± 0.005*	0.057 ± 0.002^#^	0.057 ± 0.002^#^
Total phenolics (mg/100 g)	13.3 ± 1.2	25.4 ± 2.0*	13.3 ± 1.2^#^	13.3 ± 1.2^#^
Phospholipids (mg/100 g)	8.0 ± 0.2	66.4 ± 1.5*	8.0 ± 0.2^#^	8.0 ± 0.2^#^
OSI(120°C)	7.06 ± 0.10	6.61 ± 0.12*	7.66 ± 0.25*^#^	8.54 ± 0.19*^#^
DPPH (μmol Trolox/100 g)	72.18 ± 0.51	131.71 ± 0.47*	105.41 ± 0.63*^#^	262.39 ± 0.92*^#^
FRAP (μmol Trolox/100 g)	243.64 ± 2.37	547.85 ± 2.32*	378.99 ± 3.45*^#^	968.28 ± 4.22*^#^

ND: Not Detected.

Data are given as means ± SD (n = 3). *p < 0.05 vs. SRO, ^#^p < 0.05 vs. CRO.

*SRO* Standard refined rapeseed oil, *CRO* Optimized cold-pressed rapeseed oil, *LF-SRO* Tocopherols and phytosterols restoratively added standard refined rapeseed oil, *HF-SRO* Tocopherols and phytosterols fortified standard refined rapeseed oil.

The total antioxidant capacity provided information about the duration of antioxidative action activated by certain concentration of an antioxidant or antioxidant mixture. Here, we applied the DPPH and FRAP methods to evaluate the total antioxidant capacity of different rapeseed oils. And the results indicated that the total antioxidant capacities of rapeseed oils determined by DPPH method were not similar to that analyzed by FRAP method. However, the highest correlation coefficient (r = 1.000) was observed between the DPPH and FRAP methods. The CRO, LF-, HF-SRO presented a higher total antioxidant capacities (FRAP = 547.85, 378.99 and 968.28 μmol Trolox/100 g; DPPH = 131.71, 105.41 and 262.39 μmol Trolox/100 g) than the SRO (FRAP = 243.64 μmol TE/100 g; DPPH = 72.18 μmol TE/100 g) (p < 0.05). In addition, the CRO showed the comparable total antioxidant capacity with the LF-SRO, but lower than HF-SRO, indicating that the refining process caused a significant decrease in the antioxidant property of rapeseed oil, while restorative addition or fortification of tocopherols and phytosterols significantly strengthen the antioxidant capacity of traditional refined rapeseed oil.

### Food intake and body weight

No significant differences were observed in body weight and food intake of rats among all groups (p > 0.05). There were also no significant differences in liver weight and the apparent fat digestibility (%) of the rats in SRO, CRO, LF- and HF-SRO groups (p > 0.05).

### Plasma and hepatic lipid parameters

**Figure 1:**
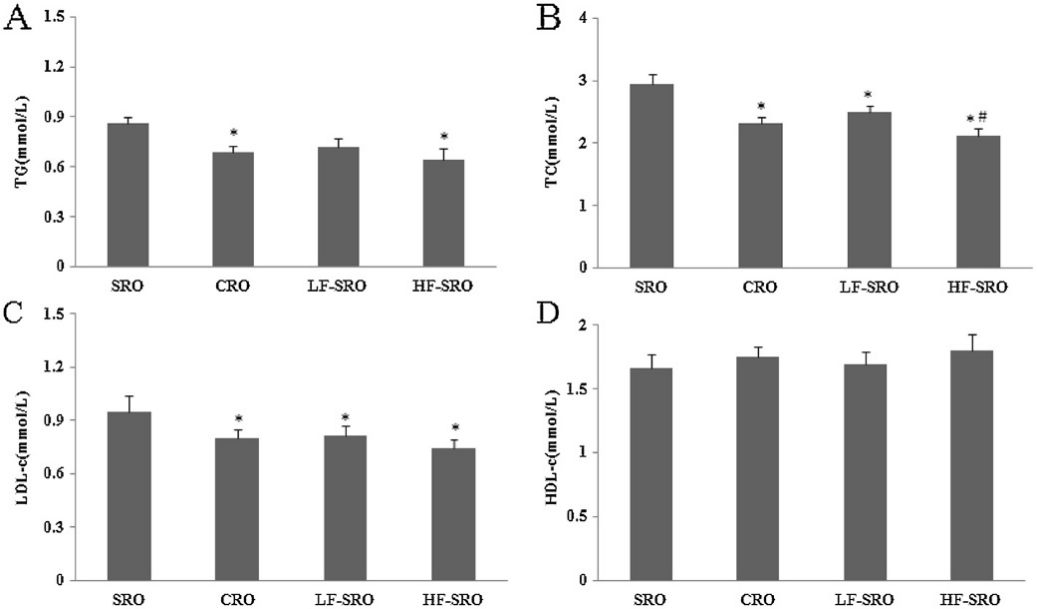
**Effect of different rapeseed oils on plasma lipid profiles of rats. (A)** plasma TG **(B)** plasma TC **(C)** plasma LDL-c **(D)** plasma HDL-c.

**Figure 2:**
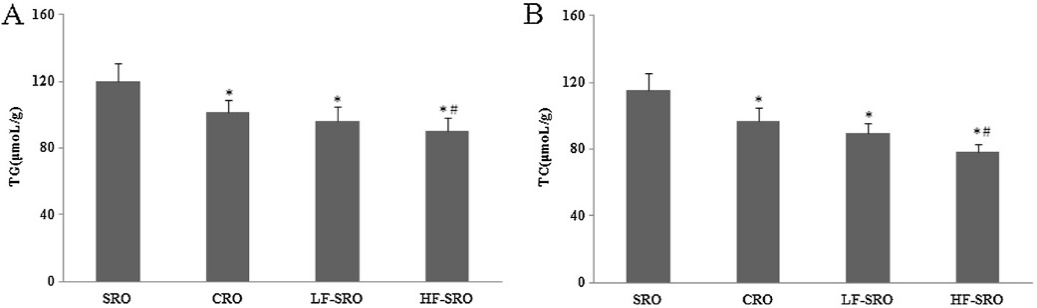
**Effect of different rapeseed oils on hepatic lipid profiles of rats. (A)** hepatic TG **(B)** hepatic TC.

The plasma lipid profiles of rats were shown in Figure 1. The plasma TG, TC, LDL-C levels of rats in CRO, LF- and HF-SRO groups decreased significantly when compared with that in SRO group (p < 0.05). No remarkable differences were revealed in the plasma TG and LDL-C levels among the CRO, LF- and HF-SRO groups, but the TC levels in the HF-SRO further decreased by 9% when compared with the CRO group. The HDL-C levels indicated no significant changes among all groups. As shown in Figure 2, the hepatic TG and TC levels of rats in CRO, LF- and HF-SRO groups decreased remarkably compared with that in SRO group (p < 0.05). In addition, significant differences in the hepatic TG, TC levels were also observed between the CRO and HF-SRO groups (p < 0.05).

### Plasma antioxidant capacity and oxidative stress

**Figure 3:**
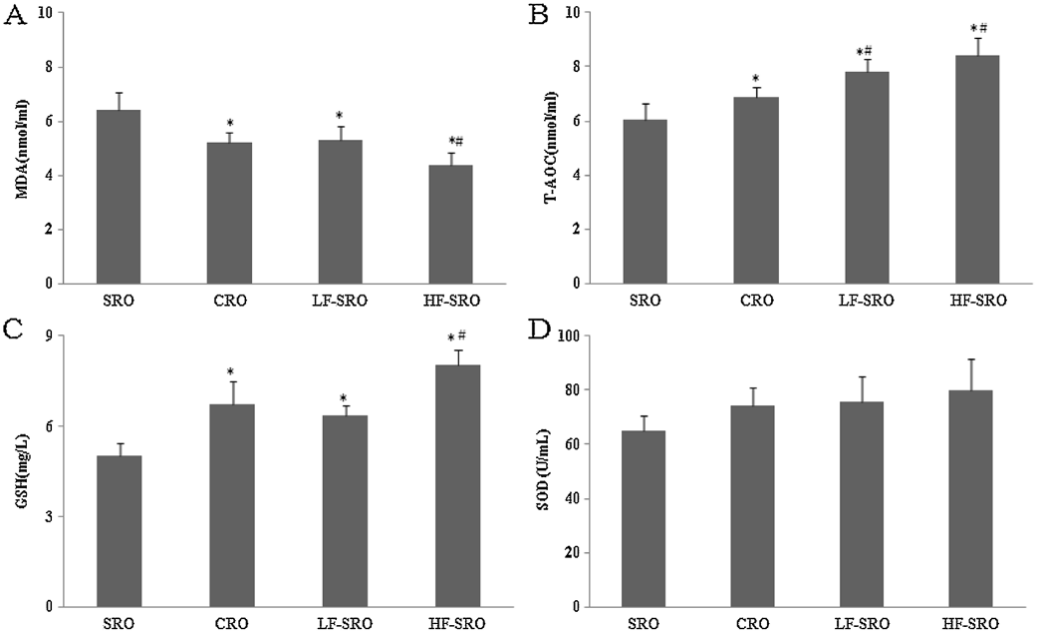
**The plasma antioxidant capacity and lipid peroxidation of rats. (A)** plasma MDA **(B)** plasma T-AOC **(C)** plasma GSH **(D)** plasma SOD.

**Figure 4:**
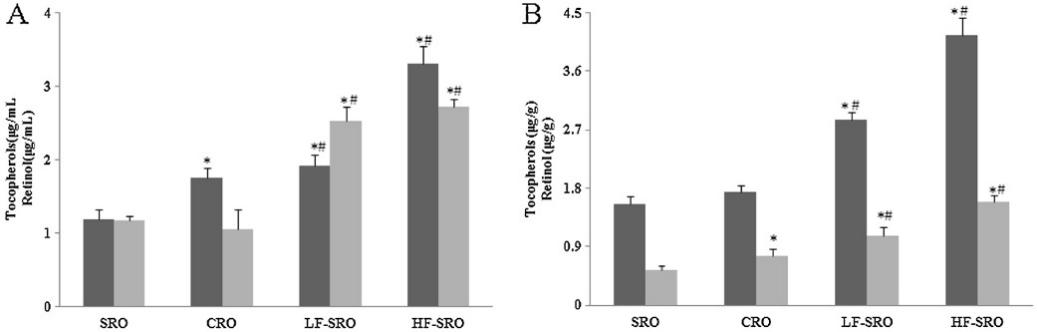
**Tocopherols and retinol contents in plasma and liver of rats. (A)** plasma tocopherols and retinol contents **(B)** hepatic tocopherols and retinol contents.

As can be seen in Figure 3, high-fat diet significantly enhanced the MDA levels and decreased T-AOC values in plasma of rats when compared with chow diet (p < 0.05). However, the isocaloric high-fat diet containing different rapeseed oil replacement partially reversed the status (unpublished data). In addition, the MDA levels in CRO, LF- and HF-SRO groups decreased by 19%, 17% and 31%, respectively, when compared with SRO group. And the rapeseed oil intervention significantly increased the plasma SOD activities and GSH levels of rats (p < 0.05), in which the rats in CRO, LF- and HF-SRO groups showed significantly higher GSH levels compared with SRO group (p < 0.05), yet the SOD activities also increased, but no significant differences were observed among these groups. The plasma and hepatic tocopherols and retinol contents were shown in Figure 4. Markedly increased plasma and hepatic tocopherols concentrations in rats fed CRO, LF- and HF-SRO were revealed in this study when compared with SRO (p < 0.05). There was also significant differences in the plasma tocopherols levels between CRO and HF-SRO groups (p < 0.05), as well as the hepatic tocopherols levels among CRO, LF- and HF-SRO groups (p < 0.05). And the plasma retinol contents in LF- and HF-SRO groups were significantly higher than that in SRO groups (p < 0.05), and significant differences in hepatic retinol contents were also observed among CRO, LF- and HF-SRO groups (p < 0.05).

### Fecal cholesterol and phytosterols

**Figure 5:**
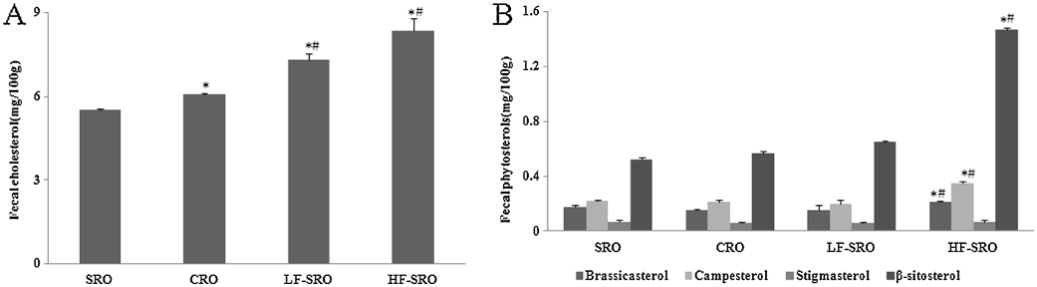
**The cholesterol and phytosterols contents in fecal of rats (mg/g dry feces). (A)** fecal cholesterol **(B)** fecal phytosterols contents.

As shown in Figure 5, the different high-fat-diet replacing rapeseed oils augmented the secretion of fecal cholesterol when compared with high-fat-diet (p < 0.05) (unpublished data), and differences also revealed between the SRO and CRO (p < 0.05). In addition, LF- and HF-SRO future increased the contents of fecal cholesterol compared with SRO and CRO (p < 0.05). The fecal total and individual phytosterols contents in rats were consistent with the intake, therefore, the highest fecal β-sitosterol, brassicasterol and campesterol contents were found in rats of HF-SRO group.

## Discussion

### The micronutrient compositions and antioxidant properties of rapeseed oils

The antioxidant properties *in vitro* of rapeseed oils with different levels of exogenous and endogenous micronutrients were assessed by using the oxidative stability index (OSI) and total antioxidant capacities (DPPH and FRAP). Compared with the traditional refined rapeseed oil, the optimized cold-pressed rapeseed oil prepared from the laboratory exhibited higher contents of tocopherols, polyphenols, phospholipids, and phytosterols, constituting the main material basis for exerting its antioxidant properties *in vitro.* Moreover, after the restorative addition or fortification of exogenous tocopherols and phytosterols, the antioxidant properties *in vitro* of traditional refined rapeseed oil were further improved. It was noteworthy that the LF-SRO supplemented with restoratively added tocopherols and phytosterols had lower total antioxidant activities than the CRO, which indicated the possibly coordinative participation of bioactive components, such as polyphenols, phospholipids and other micronutrients untested in our study, into the antioxidant properties of CRO. Tocopherols, as an antioxidant found in most of vegetable oils, physically quenched singlet oxygen, resulting in the deactivation of singlet oxygen to its ground state with no oxygen consumption or product formation. Phospholipids (PL), including phosphatidylcholine (PC) and phosphatidylethanolamine (PE), can donate hydrogen in amino group to tocopherols and synergistically decrease the autoxidation of lipids by regenerating tocopherols [8, 9]. The HF-SRO with exogenous fortified tocopherols and phytosterols, exhibited higher DPPH and FRAP values than LF-SRO and CRO. Thus, it might be more importance of their mixture than the amount of a certain antioxidant when the concentration of the exogenous added antioxidant was not dominant in the newly constructed antioxidant system in oils. While if the levels of exogenous antioxidants were higher enough, it could indicate superior antioxidant activities and exceeded the synergistic effect of endogenous antioxidants in oils. In addition, the relative lower OSI in CRO than SRO was rather unexpected and might be due to the higher water contents or other unknown factors existed in CRO due to no future refining process, and this results also revealed that the antioxidant capacity of oils may be a function of co-relationship among the presence of antioxidants, prooxidants, free fatty acid (FFA) content, peroxide value, K-values and other factors, revealing the complexity of oil antioxidant system [10].

### Effects of exogenous and endogenous micronutrients in rapeseed oils on the antioxidant status of rats

The antioxidant activity *in vivo* of different rapeseed oils showed that the CRO, as well as the LF- and HF-SRO, increased the plasma T-AOC and GSH levels and reduced the MDA levels when compared with SRO. Significant differences in plasma T-AOC, GSH and MDA levels were revealed in HF-SRO when compared with CRO and LF-SRO, which was consistent with the antioxidant capacities of CRO, LF and HF-SRO *in vitro*. The higher plasma and hepatic tocopherols contents of rats in LF- and HF-SRO groups indicated that the tocopherols were delivered to the general circulation and hepatocyte through lipid uptake pathways [11], which favorably exerted its antioxidant activity. The mechanism for relatively higher antioxidant activity *in vivo* in LF, HF-SRO might be due to either tocopherols that affected the overall resistance of lipoproteins to lipid peroxidation or the activity of PUFA in cell membranes that provide a greater mobility and thus greater membrane access to the antioxidants [12]. In addition, the comparable antioxidant activity *in vivo* between CRO and LF-SRO revealed that the higher phospholipids in CRO may also enhance the uptake of tocopherols in the intestine [13]. The higher plasma and hepatic retinol levels in rats fed LF-, HF-SRO than that in SRO and CRO may because of the higher exogenous tocopherols levels in LF-, HF-SRO, which was thought to follow the same absorptive pathway as provitamin A carotenoids in high fat diet (consisting of almost 40% chopped corn, which contained the highest levels of carotenoids among all the cereals) in our study. The mechanism might involve in the Scavenger Receptor class B type I (SR-BI), which participated in the absorption of carotenoids by the intestinal cell, but also of tocopherols. Therefore, the increased tocopherols levels in LF-, HF-SRO could enhance the absorption of carotenoids particularly in dietary dose and followed provitamin A carotenoids converting into retinol and then to retinyl esters [14, 15].

### Effects of endogenous and exogenous micronutrients in rapeseed oils on lipid profiles of rats

Many previous studies have confirmed the improved cardioprotective properties of optimized oils obtained by screw press and selective solvent extraction procedures or exogenous addition of one or more micronutrients [2, 3, 7, 16, 17]. On this basis, we future compared the effects of endogenous antioxidants in optimized cold-pressed rapeseed oil and restoratively added or fortified exogenous tocopherols and phytosterols in traditional refined rapeseed oil on the lipid-lowering activity of high-fat fed rats. And the results showed that HF-SRO favorably improved the lipid profile compared with CRO, but no significant differences were revealed between LF-SRO and CRO. In which the plasma TC levels decreased by 9% and 15% in HF-SRO group when compared with CRO and LF-SRO groups, and the hepatic TC levels simultaneously reduced by 19% and 12%, respectively. These mechanisms were related to the higher phytosterols contents in HF-SRO. The cholesterol-lowering effectiveness of phytosterols was based on the ability to reduce cholesterol absorption owing to the coprecipitation of cholesterol and phytosterols and displace cholesterol (biliary and dietary cholesterol) from micelles in the duodenum, thereby inhibiting the intestinal cholesterol absorption to compete for space in mixed micelles [18, 19]. Therefore, higher fecal cholesterol contents in HF-SRO than LF-SRO and CRO had been observed in our study. Further studies on the mechanism of LF-, HF-SRO and CRO on the cholesterol-sensors and nuclear transcription factors regulating cholesterol synthesis, esterification, and intracellular transport, and maintaining the hepatic cholesterol homeostasis should be conducted. Furthermore, high levels of tocopherols and phytosterols in LF-, HF-SRO were not accompanied by significantly changes in plasma total fatty acid composition in the present study. Some small but significant changes in phospholipid fatty acid profile were revealed, but these were not consistent between tissues or for fatty acid classes [20]. Thus, the effects of accumulation characteristics of tocopherols and phytosterols in different tissues on the fatty acid composition in different lipid classes (CE, TG, PL, and FFA) still required further investigation.

Thus, our study had showed that HF-SRO and CRO exhibited comparable roles in improving the antioxidant status and lipid profile of high-fat fed rats. Whereas, considering from the costs and feasibility, cold-pressed oil obtained from the currently optimizing refining techniques would be more suitable for promotion to the common consumers as cooking oil, yet there were strict requirements for the raw material quality of rapeseed due to no further refining processes. On the other hand, for human with the progress of cardiovascular disease, HF-SRO fortified with tocopherols and phytosterols between dietary and pharmacological concentration, would be better as a functional food for adjuvant treatment and attenuate the metabolic disorders caused by cardiovascular diseases at different stages of progress.

## Conclusions

The exogenous fortification of micronutrients (tocopherols and phytosterols) in traditional refined rapeseed oil but not merely restorative addition ameliorated the oxidative stress and favorably affected the lipid profile in high-fat fed rats, which was comparable to the optimized cold-pressed rapeseed oil and to some extent remedied the adverse effect of traditional processing technology on the biological activity of rapeseed oil.

## Materials and methods

### Materials

All rapeseed oils were produced from the same batches of rapeseeds. The standard rapeseed oil (SRO) was provided by Hulunbuir Jinjiao Bio-chemical CO., LTD. (Inner Mongolia, China) and obtained by the classical crushing steps: cooking and then pressing using a screw press and solvent extraction followed by subsequent deacidification, degumming, bleaching and deodorization. The optimized cold-pressed rapeseed oil (CRO) was achieved using the Komet Vegetable Oil expeller (Type: Oil press CA59G; Oil temperature: <60°C), and the crude oil was centrifuged, frozen at 4°C for 24 h, and then centrifuged again. The SRO contained 45.5 mg tocopherols and 686 mg phytosterols per 100 g oil, respectively; the CRO had 51.0 mg tocopherols and 920 mg phytosterols per 100 g oil, respectively; the restoratively added or fortified rapeseed oils (LF-, HF-SRO) contained 100, 1000 and 200, 2000 mg tocopherols and phytosterols per 100 g oil, respectively. Specific methods were performed as follows: A specific amount of traditional refined rapeseed oil and tocopherols/phytosterols was weighed, well-mixed at 60°C of water bath for 30 min, and then stored at 4°C after cooling.

Tocopherols (purity ≥ 50%, α: 13.32%, β + γ: 30.70%, δ: 8.25%) were obtained from Wuhan Yuancheng Gongchuang Technology CO., LTD. Phytosterols (purity ≥ 95%, β-sitosterol ≥ 70%) were obtained from Xi′an Lantian Biological Technology Co., LTD. α, γ–Tocopherols (purity > 96%), 5α-cholestane (purity > 97%), Folin–Ciocalteu reagent,2,4,6-tris(2-pyridyl)-s-triazine (TPTZ, purity > 99%), 6-hydroxy-2,5,7,8-tetramethylchroman-2-carboxylic acid (Trolox, purity > 97%), 2,2-diphenyl-1-picrylhydrazyl radical (DPPH, purity > 95%) were purchased from Sigma–Aldrich (Saint Louis, MS, USA). Fatty acid methyl ester standards were purchased from NU-CHEK-PREP.

### Determination of physicochemical and antioxidant properties of different rapeseed oils

#### Analysis of physicochemical properties

The acid value (AV) and peroxide value (PV) of SRO, CRO, LF- or HF-SRO were determined according to the methods from ISO 3960:2007 and ISO 660:1996. The water and volatile matter and phospholipids contents were determined according to the National Standard of the PRC (GBT 5528-2008, GB/T 5537-2008). Fatty acid methyl ester were prepared according to the method from ISO 12966-2:2011 and analyzed using an Agilent 6890 GC with a Flame Ionization Detector (FID) and a 30 m × 0.32 mm × 0.25 μm HP-INNOWAX fused silica capillary column. Oven temperature was programmed from 210°C for 9 min to 230°C at 20°C/min and held for 10 min. N_2_ was the carrier gas at 1.5 mL/min. Injection volume was 1 μL at a split ratio of 80:1. The individual fatty acid was identified by comparison with commercial standards.

#### Analysis of oxidative stability

The oxidative stability index (OSI) was determined with a Metrohm Rancimat model 743 according to method from Farhoosh [21]. Briefly, oil sample (3.0 g) contained in a reaction vessel was placed in an electric heating block. Filtered, cleaned, dried air was allowed to bubble through the hot oil under an flow of 10 L/h. Effluent air containing volatile organic acids from the oil sample were collected in a vessel containing 60 mL of distilled water. The conductivity of the water was measured automatically as oxidation proceeded. And the OSI was automatically recorded at 110°C and expressed in hours (h).

#### Analysis of total antioxidant capacities

##### Extraction of antioxidants

The extracts of antioxidants from rapeseed oil were obtained according to Szydlowska-czerniak et al [6]. Briefly, a test tube with oil (5 g weighed with the precision of 0.0001 g) and methanol (5 mL) were shaken for 30 min at room temperature in the dark. And the extracts were separated from the oil by centrifugation at 5000 rpm for 10 min and quantitatively transferred into glass bottles. Each sample was extracted three times using methanol. All extracts were stored prior to analysis in dark glass bottles in a refrigerator set below 4°C.

##### DPPH method

The 2, 2-Diphenyl-1-picrylhydrazyl (DPPH) method described previously was used to determine the total antioxidant capacity of different rapeseed oils [22]. Briefly, 0.5 mL of the methanol extracts was added to 2.5 mL DPPH methanol solution (38 μg/mL). The mixture was shaken vigorously and left in the dark for 30 min. The absorbance was measured at 515 nm against pure methanol (blank) using a UV-1800PC spectrophotometer (Shanghai, China) in a 1 cm quartz cell. The scavenging of DPPH was calculated as follows:%DPPH scavenging = [(A_control_ -A_sample_)/A_control_] × 100%, yet the DPPH values were expressed in μM Trolox equivalents per 100 g oil.

##### FRAP method

The antioxidant capacity of different rapeseed oils was determined using the ferric reducing antioxidant power (FRAP) method [23]. Briefly, freshly prepared FRAP reagent (2.5 mL of a 10 mM TPTZ solution in 40 mM HCl, 2.5 mL of 20 mM FeCl_3_ and 25 mL of 0.1 M acetate buffer, pH 3.6) was incubated at 37°C for 10 min. Next, 1.0 mL of methanol extracts and 2 mL of FRAP reagent were transferred into a 10 mL volumetric flask containing distilled water. The obtained blue solutions were maintained at room temperature for 20 min. The absorbance was measured at 593 nm against a reagent blank (2 mL of FRAP reagent made up to 10 mL with redistilled water) using the UV-1800PC spectrophotometer (Shanghai, China) in a 1 cm quartz cell.

#### Analysis of total phenolics, phytosterols and tocopherols

The total phenolics were determined using the Folin–Ciocalteau colourimetric method according to Koski et al [24]. Briefly, 0.5 mL of the extracts was transferred to a 25 mL colorimetric tube. Next, 1 mL of Folin–Ciocalteu phenol reagent and 3 mL of sodium carbonate solution (20%) were added and brought up to 25 mL by adding distilled water. After 1 h, the absorbance was measured at 765 nm using a DU 800 UV/Visible Spectrophotometer (Beckman Coulter Inc., USA). Gallic acid was used for the calibration, and the results of duplicate analyses were expressed as mg gallic acid/100 g flaxseed. The phytosterols were analyzed according to a modified method described by Azadmard-Damirchi et al [25]. In brief, the 0.03 g different rapeseed oils (exact to 0.0001 g) was mixed thoroughly with 150 μL 5α-cholestane (0.5 mg/mL) and 3 mL of 2 mol/L NaOH/ethanol in a glass tube, then capped and heated in a shaking water bath at 90°C for 15 min. The samples were cooled to room temperature, and 2 mL water and 1.5 ml hexane were then added to the solvent, followed by mixing and centrifugation at 1500 × *g* for 10 min. The upper hexane layer was collected and dried under N_2_. Next, the residue was derivatized with 100 μL Tri-Sil under ultrasonic for 30 min and reconstituted in 1.5 mL hexane after drying under N_2_. Phytosterols were separated using an Agilent 6890 GC with a flame ionization detector and a 30.0 m × 0.32 mm × 0.10 μm DB-5HP fused silica capillary column. The injector and detector temperatures were set at 260°C and 310°C, respectively. The initial column temperature was 60°C and increased to 310°C at 40°C/min and held for 6 min. N_2_ was used as a carrier gas (2.0 mL/min). The injection volume was 1 mL, and the split ratio was 25:1. The individual phytosterol was identified by comparing with authentic standards and quantified relative to the 5α-cholestane. Tocopherols contents were analyzed according to the National Standard of the PRC (GB/T 5009.82-2003) using Ultra-High-Pressure Liquid Chromatography (UPLC). Approximately 0.1 g of oil samples (exact to 0.0001 g) was accurately weighed, and then 1 mL of ascorbic acid (0.05 g/mL), 5 mL of anhydrous ethanol and 0.5 mL of KOH solution (14.26 mol/L) were added and saponified at 70°C for 30 min. After cooling, 3 mL of distilled water was added to the solvent, and the suspension was extracted twice with 5 mL of hexane/ethylacetate (85:15, v/v) followed by mixing and centrifugation at 1500 × *g* for 15 min. The hexane phase was collected and dried under N_2_. Next, the residue was reconstituted in the mobile phase (100 μL), mixed well and centrifuged at 3500 rpm for 5 min and injected for Ultra Performance Liquid Chromatography (UPLC) analysis (Waters, Milford, MA, USA). The separation was performed on a Zorbax Eclipse XDB-C18 column (100 mm × 2.1 mm diameter and 1.8 mm particles, Agilent Technologies, Palo Alto, CA, USA). The Mobile phase was acetonitrile/methanol/ethyl acetate (60:20:20, v/v/v) delivered at a flow rate of 0.30 mL/min. The monitoring wavelengths were 300 nm and the injection volume was 2 μL. Tocopherols was quantified using an external standard method with respective reference samples [26].

#### Animals and diets

Male Wistar rats weighing 150-160 g were obtained from Vital River Laboratory Animal Center (Beijing, China). The animals were housed in stainless steel cages at a controlled room temperature of 20 ± 1°C with a 12:12 light: dark cycle. The animals were cared for in accordance with the Guiding Principles in the Care and Use of Animals. After one week of acclimatization with chow diet, the rats were switched to different isocaloric high-fat diets containing 20% fat for 4 weeks. The high fat diets contained 82.7% chow diet (containing 5% fat), 16% lard with 10% SRO, CRO, LF-SRO, HF-SRO replacement, 1% cholesterol and 0.3% bile salts. The chow diet was provided by the Experimental Animal Center of Disease Control and Prevention Center of Hubei Province. The animals were given free access to food and water. All diets were prepared weekly and stored at –80°C.

Feed and water were changed every day, and the body weights of the rats were recorded twice per week. Daily feed (g) was calculated on a per rat daily basis. At the end of the experiment, the rats were anesthetized using 0.2% pentobarbital sodium and then sacrificed. Blood was collected in 0.2% EDTA, and plasma was obtained by centrifugation 1500 × *g* for 15 min at 4°C, and then stored at –80°C. The liver from each rat was excised, washed in ice-cold saline, weighed and stored at –80°C for further analysis.

#### Analysis of apparent fat digestibility

The feces excreted during the last 3 days of the experimental period were collected and stored at –80°C after vacuum drying at 60°C. And the fecal samples were pulverized and weighed prior to analysis. Fecal lipids were determined gravimetrically using a modification of the Saxon method. The apparent fat digestibility was obtained using the equation of [(intake of lipid-fecal lipid)/intake of lipid × 100] [27].

#### Analysis of plasma and hepatic lipid parameters

Plasma total cholesterol (TC), triglyceride (TG), low-density lipoprotein cholesterol (LDL-C) and high-density lipoprotein cholesterol (HDL-C) were determined by enzymatic colorimetric methods using commercial kits (Biosino Bio-Technology and Science Inc., Beijing, China). These methods were based on the detection of colored end-products at 500 nm.

Liver lipids were extracted as previously described by Folch et al [28]. Briefly, liver tissue (0.1–0.2 g) was homogenized in chloroform/ methanol (2/1, v/v) to a final volume of 20 times the tissue sample. The homogenate was filtered with Whatman No.1 filter paper to obtain the liquid phase. The extract was then dried under N_2_ and resuspended in isopropanol. Hepatic TC and TG levels were measured using the same enzymatic commercial kits as the plasma analysis.

#### Analysis of plasma antioxidant capacity and lipid peroxidation

The T-AOC level was determined using the Fe^3+^ reduction method according to the commercial test kits (Nanjing Jiancheng Bioengineering Institute, Nanjing, China). The SOD activity was estimated basing on the xanthine-xanthine oxidase-nitrite method according to Fridovich with slight modification [29]. The GSH content was estimated by the use of commercial kit (Nanjing Jiancheng Bioengineering Institute, China) based on the method of Eady et al [30]. MDA levels were measured using the thiobarbituric acid (TBA) method [31], which combines with MDA and forms a pink chromogen, where the absorbance at 532 nm was measured. MDA levels were expressed as TBARS in nanomoles per milliliter plasma.

#### Analysis of plasma and hepatic fat-soluble vitamins

Frozen plasma samples were kept at 4°C in the dark prior to analysis. The assay of fat-soluble vitamins was based on a modified method from Paliakov et al [32]. Briefly, 200 μL of plasma was deproteinized in 0.5 mL methanol and then mixed by vortexing and extracted twice with 0.75 mL hexane for 5 min. The sample was centrifuged at 1500 × *g* for 15 min. Determination of hepatic fat-soluble vitamins was performed as described by Schmitz et al [33]. Approximately 1 g of liver tissue was homogenized in 5 mL saline, and then 3 mL of NaOH/ethanol solution (2.0 mol/L) was added and saponified at 100°C for 30 min. After cooling, 5 mL of distilled water and 1 mL hexane were added to the solvent, followed by mixing and centrifugation at 1500 × *g* for 15 min. The hexane phase was collected and dried under N_2_. Next, the residue was reconstituted in the mobile phase (50 μL), mixed well and transferred to an amber sample vial for Ultra Performance Liquid Chromatography (UPLC) analysis (Waters, Milford, MA, USA).

The separation was performed on a ZORBAX Eclipse XDB-C18 column (2.1 mm I.D.100 mm length, 1.8 μm particle size) maintained at 40°C. The mobile phases consisted of acetonitrile (mobile phase A) and methanol/ethyl acetate (1:1, mobile phase B) delivered at a flow rate of 0.3 mL/min, A: B = 60:40, and the injection volume was 2 μL.

#### Analysis of fecal cholesterol and phytosterols

Fecal samples (0.5 g) were ground to a fine powder and extracted twice with 20-fold weight to volume ratio of chloroform/methanol (2/1, V/V) in a screw-capped glass tube. The extract was filtered using Whatman No.1 filter paper and dried under N_2_. Next, 100 μL 5α-cholestane (0.255 mg/mL) and 3 mL of 0.5 N NaOH/ethanol were added to each sample, which were then capped and heated at 100°C for 30 min. The samples were cooled to room temperature, and 3 mL water and 1 ml hexane were then added to the solvent, followed by mixing and centrifugation at 1500 × *g* for 15 min. The upper hexane level was analyzed using capillary gas chromatography described previously [34, 35]. Fecal cholesterol and phytosterols were separated using an Agilent 6890 GC with a flame ionization detector and a 100 m × 0.25 mm DB-1 fused silica capillary column. The injector and detector temperatures were set at 260°C and 330°C, respectively. The initial column temperature was 220°C and increased to 300°C at 2°C/min and held for 10 min. N_2_ was used as a carrier gas (1.5 mL/min).

#### Statistical analysis

The values were presented as the mean ± SD. The statistical significance of the differences between groups was determined by one-way ANOVA followed by Tukey's test using SPSS statistical software. Differences among groups were considered significant at p <0.05*.*

## Abbreviations

CVD: Cardiovascular disease
TG: Total triglyceride
TC: Total cholesterol
LDL-C: Low-density lipoprotein cholesterol
HDL-C: High-density lipoprotein cholesterol
CE: Cholesteryl ester
FFA: Free fatty acid
PL: Phospholipid
SRO: Standard refined rapeseed oil
CRO: Optimized cold-pressed rapeseed oil
LF-SRO: Tocopherols and phytosterols restoratively added standard refined rapeseed oil
HF-SRO: Tocopherols and phytosterols fortified standard refined rapeseed oil
SOD: Superoxide dismutase
MDA: Malondialdehyde
GSH: Glutathione
T-AOC: Total antioxidant capability
PUFAs: polyunsaturated fatty acids
DPPH: 1, 1-diphenyl-2-picryl-hydrazyl
FRAP: Ferric-reducing antioxidant power
EDTA: Ethylene Diamine Tetraacetic Acid.
